# Sharpening the focus on gaming disorder

**DOI:** 10.2471/BLT.19.020619

**Published:** 2019-06-01

## Abstract

The definition of gaming disorder is an important first step in developing a public health response to a new problem. Gary Humphreys reports.

Dr Susumu Higuchi has no doubt about the mental health risks posed by on-line gaming.

He heads the Kurihama Medical and Addiction Centre in Kanagawa Prefecture, Japan, which started the country’s first programme for internet addiction in 2011. There are now 84 nationwide. Higuchi has watched the number of patients addicted to on-line gaming steadily grow.

“Of the 269 patients we now see for internet addiction, 241 have gaming disorder as their principal addiction”, he says. “Of those, 215 are males.”

The patients Higuchi sees display a range of symptoms, but are generally unable to limit the time they spend gaming and continue to play despite negative consequences, such as dropping out of school (almost three quarters of the patients are students) or losing a job.

No national survey of gaming disorder has been undertaken in Japan. However, a recent national survey of the broader category of “internet addiction” reported that approximately 1.82 million males 20 years of age and older, were living with an internet addiction in 2018, almost three times the number reported in 2013. The same survey reported 1.3 million adult females living with internet addiction, up from 0.5 million in 2013.

Higuchi co-authored a recent literature review - *Cross-sectional and longitudinal epidemiological studies of internet gaming disorder* – that found a prevalence of internet gaming disorder in the samples reviewed ranging from 0.7% to 27.5%.

“The literature review revealed that geographical region made little difference to prevalence,” says Vladimir Poznyak, an expert on substance use and addictive behaviours at the World Health Organization (WHO), who points to several surveys showing internet gaming disorder prevalence between 1%-10% in Europe and North America.

“Because of differences in survey quality and comparability, the exact size and nature of that problem is yet to be defined,” he says, “but it is clear there is a problem.”

In Switzerland, a report commissioned by the Federal Office of Public Health published in 2018 found that around 1% of the population (approximately 70 000 people) are “problematic” internet users.

One of the experts consulted for that report – Dr Sophia Achab – runs a behavioural addiction programme at the University Hospital of Geneva where, since 2007, she has been treating patients for internet-use disorders ranging from addiction to on-line gambling to internet pornography.

“Addiction to gaming is harder to treat than addiction to alcohol or drugs because the internet is everywhere.”

Susumu Higuchi

Like Higuchi, Achab has seen a steady increase in on-line gaming disorder patients, as well as an increasing proportion of younger, male patients. “Today, 43 of our 110 patients with internet addiction are primarily addicted to gaming, 40 of them boys and young men, and just three girls,” she says.

Among the people that have left the deepest impression on Achab, was a 22-year-old man who was brought in by his mother. “He had dropped out of school two years earlier and refused to leave his room where he played for 18 hours a day. He was suffering blood clots in his legs due to physical inactivity,” she says.

Treating such patients is extremely challenging, partly because of the ubiquity of the internet. “In some ways addiction to gaming is harder to treat than addiction to alcohol or drugs because the internet is everywhere,” says Higuchi.

Another challenge is the way the games themselves are designed.

The nature of the game played is one of the three factors considered by Achab in her assessment of patient exposure to addiction risk, the others being individual factors, such as self-esteem, and environmental factors, such as the home, school or work environment.

For Achab, the presence of reward systems (often mediated through virtual ‘loot boxes’) offering virtual items such as weapons and armour or ‘real’ rewards, such as video streaming subscriptions, are red flags. “Rewards drive players to rack up the hours in pursuit of virtual or real-world gains,” she explains.

For Higuchi, multiplayer on-line games are also a matter of concern. “Such games provide opportunities to play and compete with others, which would be compelling for most people, but particularly for those who might otherwise find it hard to socialize,” he says.

Higuchi also points to games that encourage players to compete in tournaments and competitions for prize money. “Many of my patients talk about making a living from game play,” he says. “This belief feeds into the broader pathology.”

Approaches to treating those with on-line gaming disorder tend to focus on getting the patient to recognize their addiction and to reconnect them with reality. Higuchi uses a mix of cognitive behavioural therapy, social skills development, and treatment programmes emphasising physical activity. Achab uses psychotherapy to reconnect patients with themselves, their life objectives and their social environment.

To date, the task of clinicians has been made harder by the lack of consensus regarding the nature of the condition they are treating. “The lack of clarity around the definition of gaming disorder not only makes it harder to develop appropriate treatment and public health policy, it also stands in the way of effective monitoring and surveillance,” Higuchi says.

It was partly to address this issue that WHO initiated a four-year consultation process to explore public health implications of gaming and establish clear boundaries for ‘gaming disorder’. The classification derived from that consultation was published in the 11^th^ edition of the *International statistical classification of diseases and related health problems* (ICD-11) the diagnostic classification standard used by health professionals ranging from hospital administrators to clinicians and researchers. 

According ICD-11, a diagnosis of gaming disorder is appropriate for a person who, over a period of at least 12 months, lacks control over their gaming habits, prioritizes gaming over other interests and activities, and continues gaming despite its negative consequences.

“The inclusion of gaming disorder in ICD-11 will facilitate appropriate diagnosis and treatment.”

Vladimir Poznyak

The decision to establish a new diagnostic category and include it in ICD-11 has been welcomed by psychologists and psychiatrists worldwide, including members of the Royal College of Psychiatrists in the United Kingdom of Great Britain and Northern Ireland and Division 50 of the American Psychological Association (APA) – the division focused on addiction psychology.

Not everyone is happy, however. Gaming industry associations and some mental health professionals and academics have argued that, given the current state of knowledge regarding the impact of gaming on individuals, the inclusion is premature, and is likely to lead to overdiagnosis while also feeding into moral panic about on-line gaming and stigmatization of gamers.

Critics making these arguments cite the APA’s decision to enter ‘internet gaming disorder’ as a ‘condition for further study’, in the 2013 *Diagnostic and Statistical Manual of Mental Disorders* (DSM-5), a designation signifying that further research is required before it can be accepted as a valid diagnostic category.

WHO’s Pozynak points out that the inclusion of gaming disorder in ICD-11 was based on the conclusions of experts from more than 20 countries, as well as evidence of increasing internet-gaming-related treatment demand. 

As for concerns regarding over-diagnosis and stigmatization, Poznyak is sceptical. “The inclusion of gaming disorder in ICD-11 will facilitate appropriate diagnosis and treatment as well as the monitoring, surveillance and research required to get a clearer picture of the prevalence and impact of the condition,” he says, adding that WHO is currently working with partners on the development of an evidence-based screening and diagnostic interview to support clinicians.

According to Dr Charles O'Brien, Professor of Psychiatry at the University of Pennsylvania, and chair of the APA committee that decided to include internet gaming disorder in DSM-5 under the ‘condition for further study’ rubric, the classification is currently under review.

“There have been a lot of developments since 2013, and we have the option to change the disorder classification if we consider it appropriate,” says O’Brien. 

Higuchi welcomes any move towards clearer diagnosis, and greater recognition of the disorder. “The ICD-11 classification will help with that,” he says. He also welcomes WHO’s decision to publish guidelines on physical activity for children under 5 years of age, which recommend, among other things, that children in their first year of life should have no screen time and very little in their second, while those aged 2 to 4 years, should spend no more than an hour a day in front of a screen. 

“It’s time to set limits,” Higuchi says.

**Figure:**
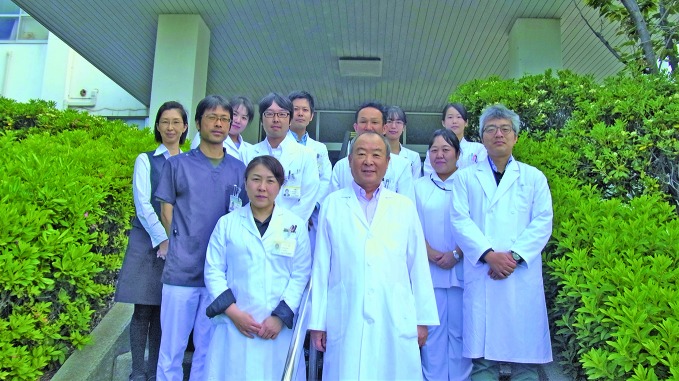
Staff at the Kurihama Medical and Addiction Centre, Kanagawa Prefecture, Japan.

Courtesy of Kurihama Medical and Addiction Centre

**Figure:**
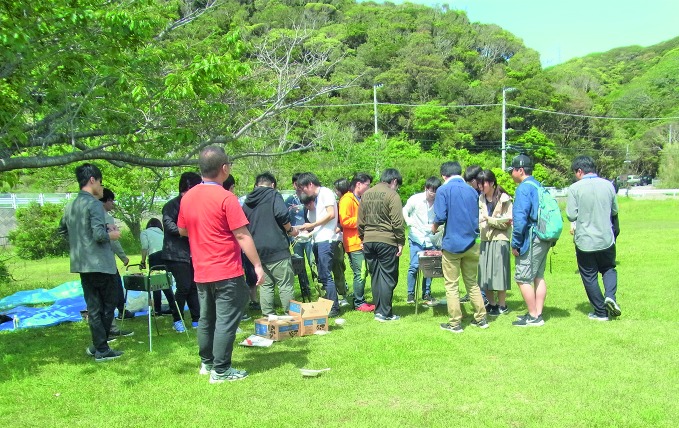
Patients undergoing treatment for gaming disorder socialize as part of their therapy, Kurihama Medical and Addiction Centre.

